# The Nuclear Receptor NR4A1 Induces a Form of Cell Death Dependent on Autophagy in Mammalian Cells

**DOI:** 10.1371/journal.pone.0046422

**Published:** 2012-10-05

**Authors:** Jimena Bouzas-Rodríguez, Gabriela Zárraga-Granados, Maria del Rayo Sánchez-Carbente, Rocío Rodríguez-Valentín, Xicotencatl Gracida, Dámaris Anell-Rendón, Luis Covarrubias, Susana Castro-Obregón

**Affiliations:** Developmental Genetics and Molecular Physiology Department, Instituto de Biotecnología, Universidad Nacional Autónoma de México, Cuernavaca, Morelos, México; Istituto Nazionale per le Malattie Infettive, Italy

## Abstract

The control of cell death is a biological process essential for proper development, and for preventing devastating pathologies like cancer and neurodegeneration. On the other hand, autophagy regulation is essential for protein and organelle degradation, and its dysfunction is associated with overlapping pathologies like cancer and neurodegeneration, but also for microbial infection and aging. In the present report we show that two evolutionarily unrelated receptors—Neurokinin 1 Receptor (NK_1_R,) a G-protein coupled receptor, and Insulin-like Growth Factor 1 Receptor (IGF1R), a tyrosine kinase receptor—both induce non-apoptotic cell death with autophagic features and requiring the activity of the autophagic core machinery proteins PI3K-III, Beclin-1 and Atg7. Remarkably, this form of cell death occurs in apoptosis-competent cells. The signal transduction pathways engaged by these receptors both converged on the activation of the nuclear receptor NR4A1, which has previously been shown to play a critical role in some paradigms of apoptosis and in NK_1_R-induced cell death. The activity of NR4A1 was necessary for IGF1R-induced cell death, as well as for a canonical model of cell death by autophagy induced by the presence of a pan-caspase inhibitor, suggesting that NR4A1 is a general modulator of this kind of cell death. During cell death by autophagy, NR4A1 was transcriptionally competent, even though a fraction of it was present in the cytoplasm. Interestingly, NR4A1 interacts with the tumor suppressor p53 but not with Beclin-1 complex. Therefore the mechanism to promote cell death by autophagy might involve regulation of gene expression, as well as protein interactions. Understanding the molecular basis of autophagy and cell death mediation by NR4A1, should provide novel insights and targets for therapeutic intervention.

## Introduction

NR4A1 (also known as Nur77, TR3, and NGFI-B, among other designations) is an orphan nuclear receptor member of the thyroid/steroid receptor superfamily, whose activity and intracellular localization is regulated by phosphorylation, and which plays a role in cell fate decisions [1]. NR4A1 was originally characterized as an immediate early response gene, as it is transiently induced in response to mitogenic factors in quiescent fibroblasts. Despite this initial designation, in response to all-trans retinoic acid NR4A1 may mediate the arrest of cells at the G_0_/G_1_ phase of the cell cycle [2]. NR4A1 expression is induced by multiple stimuli in different types of cells. Interestingly, this nuclear receptor is also involved in the regulation of cell death induced by stimuli as variable as engagement of the T cell receptor during T cell negative selection, in prostate and lung cancer cells exposed to chemotherapeutic drugs (synthetic retinoid CD437) [3], in macrophage apoptosis [4], or in thapsigargin-induced cell death [5]. Paradoxically, NR4A1 has also been reported to act as a death inhibitory factor, blocking cell death induced by ceramide [6] or by tumor necrosis factor [7]. Therefore, the underlying mechanisms that determine which fate a cell will follow upon NR4A1 activation clearly deserve investigation. Among the different possible outcomes, we are particularly interested in cell death.

Physiological cell death is fundamental to counter cell division and to eliminate harmed cells. During metazoan development, programmed cell death is essential to sculpt organs and to correct erroneous cell migration. While apoptosis is the most common program of cell death, other mechanisms also occur [8], [9], [10]. Mice lacking key apoptotic effectors such as Apaf-1, Bax, Bak, or executioner caspases −3 and −9 show minor developmental abnormalities and reach adulthood [11]. Therefore, alternative, non-apoptotic pathways for cell death may contribute to proper development. Indeed, the death of linker-cells during *C. elegans* development is independent of the apoptotic machinery, and instead requires a polyglutamine-repeat protein named Pqn-41 [12]. Also, the regression of salivary glands [13] and midgut [14] in Drosophila metamorphosis is mediated by autophagy genes.

Autophagy is a catabolic process every cell undergoes to recycle long-lived proteins and to eliminate damaged macromolecules and organelles; therefore, it helps to maintain the cells' health [15]. It is induced by nutrients and growth factors limitation, allowing the cell to survive for longer periods by recycling components. Autophagy also helps to prevent neurodegeneration by degrading missfolded proteins [16]. However, under some circumstances autophagy promotes cell death by an unknown mechanism. Among the different mechanisms of autophagy, macroautophagy (here referred to as autophagy) is the major one associated to cell death. This type of autophagy is characterized morphologically by the engulfment of cytoplasmic constituents, wrapped by double or multiple membrane sacs called autophagosomes. Lysosomes fuse to the autophagosomes to degrade the cytoplasm-derived contents and the inner membrane.

A form of cell death is defined as autophagic when, in addition of displaying autophagic features, inhibition of the autophagic core machinery prevents it. For example, L929 mouse fibroblasts undergo cell death by autophagy (hence autophagic cell death) triggered by death receptors signaling, since the cell death is dependent on the function of Atg7 and Beclin-1, while the inhibition of caspase-8 during such signaling, although preventing apoptotic cell death, allows a vesiculated cell death [17].

Previously we described a non-apoptotic form of cell death activated by the G-protein coupled receptor of Neurokinin 1 (NK_1_R) upon ligand engagement by substance P (SP) [18]. NK_1_R signaling plays a role in numerous biological processes, such as the transmission of pain in the spinal cord [19]. The cell death induced by NK_1_R/SP is characterized by cytoplasmic vacuolation, lack of caspase activation, lack of inhibition by caspase inhibitors and by Bcl-x_L_, lack of nuclear fragmentation or membrane blebbing, lack of phosphatidylserine exposure and a requirement for new gene transcription and translation. It is worth to highlight that this form of cell death is activated in apoptotic competent cells [18]. Whether the cytoplasmic vacuolation is related to autophagy has not been evaluated before. The molecular pathway activated by NK_1_R/SP to promote cell death involves a MAPK signaling cascade initiated by Raf-1, and including specifically MEK2 and ERK2 activity, but not MEK1 or ERK1 [20]. Among the genes induced in response to SP, NR4A1 is of particular interest since it is phosphorylated by ERK2 but not ERK1 [21], and because its induction has been observed in situations in which neuronal death occurs, such as kainic acid-induced seizures [22] and ischemic brain injury [23]. Moreover, in these paradigms, SP signaling is also necessary for neural death [24], [25], [26]. We found that during SP-induced non-apoptotic cell death ERK2 leads to phosphorylation of NR4A1, and that NR4A1 function is essential, since its inhibition by either dominant negative mutants or by RNAi prevents cell death [20].

Interestingly, expression of the tyrosine kinase receptor IGF1R (insulin-like growth factor 1 receptor) induces a non-apoptotic form of cell death dubbed paraptosis [27], which, in addition to resembling morphologically NK_1_R/SP-induced death, proved to be mediated also by MEK2 but not MEK1 [28]. IGF1R is widely expressed in the body, including within the brain. Upon IGF1 binding, IGF1R undergoes auto-phosphorylation, associates with intracellular adaptor proteins called IRSs (insulin/IGF1 receptor substrates), and activates either the Akt or the Ras/MEK/ERK signaling pathways (reviewed in [29]). The contribution of autophagy to paraptosis has not been addressed previously.

Taking together the MEK2 requirement in both of these non-apoptotic cell death paradigms, and the role of NR4A1 as an ERK2-specific substrate, we hypothesized that the death signaling cascades mediated by NK_1_R/SP and IGF1R converge on NR4A1 to promote a similar death program. Since in both cases vesiculated death has been observed, perhaps the autophagic machinery is exploited to produce the vesicles (even though these paradigms have previously been shown to be more similar morphologically to type III than type II pcd). In the present work we found, indeed, that the expression of NR4A1 dominant negative mutants inhibited non-apoptotic cell death in both of these paradigms. Also, we observed autophagic features such as LC3-II protein processing and redistribution, and autophagosomes by ultrastructure electron microcopy analysis. Downregulation of the pro-autophagic genes PI3K-III, Beclin-1 or Atg7 inhibited both IGF1R- and NK_1_R/SP-induced death. We then inhibited the NR4A1 family in a canonical model of cell death by autophagy, induced by the presence of a caspase inhibitor in mouse fibroblast L929 cells [17], and we observed significant cell death protection. Therefore, NR4A1 acts as a modulator of cell death that depends on autophagy induced by three different stimuli in different cell types. To gain insight into the mechanism of NR4A1 to promote this form of cell death, we examined its intracellular localization. Although most of the cells displayed NR4A1 in the nucleus, 14% of the cells showed it also in the cytoplasm. Using a luciferase reporter gene we found that NR4A1 is transcriptionally competent during cell death, suggesting that NR4A1 could acts at least in part by regulating gene transcription. Considering that the cytoplasmic fraction could also contribute to cell death, potential NR4A1 interaction partners were evaluated. We found that NR4A1 did not interact with Beclin-1 complex, although it interacted with the tumor suppressor p53, which is an autophagy regulator [30]. Because NR4A1 has previously been shown to be a mediator of apoptotic cell death, as well, we propose that NR4A1 is a key regulator of cell fate, mediating both apoptotic and non-apoptotic forms of cell death.

## Results

### IGF1R induces non-apoptotic cell death through NR4A1 activation

Considering the morphological resemblance between NK_1_R/SP [18]- and IGF1R-induced death [27], and the activation of the same MAPK pathway (namely MEK2), we tested the requirement of NR4A1 activity for non-apoptotic pcd induced by IGF1R.

**Figure 1:**
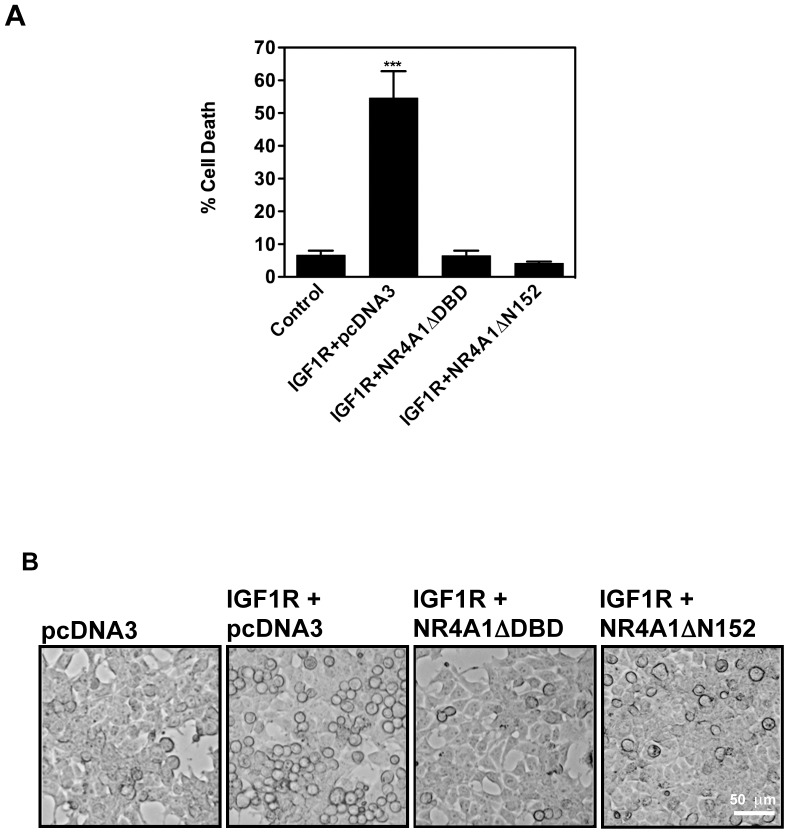
Expression of NR4A1 dominant negative mutants inhibits IGF1R-induced death. HEK293 cells were co-transfected with the indicated plasmids. Co-expression of dominant negative mutants NR4A1ΔDBD or NR4A1ΔN152 significantly reduced cell death. **A**, Percentage of cell death was determined by trypan blue exclusion 24 hr after transfection. Bars indicate standard error. ***, p<0.001, n = 4. **B**, Morphology of the rescued cells observed by phase contrast microscopy.

NR4A1 displays multiple pro-apoptotic mechanisms, including transcription of pro-apoptotic genes, translocation to the mitochondria [31], and translocation to the endoplasmic reticulum [32] to modulate the function of Bcl2 family members [33]. These actions lead to cytochrome c release from mitochondria and caspase activation. Two different NR4A1 dominant negative mutants inhibit apoptosis: one mutant lacks the DNA binding domain (NR4A1ΔDBD), and inhibits its transcriptional activity, whereas the other one lacks the trans-activating domain (NR4A1ΔN152), and inhibits NR4A1 translocation to mitochondria [31] (although the latter mutant may also prevent transcriptional activity). We observed that both mutants significantly reduced IGF1R-induced death (Fig. 1), suggesting that NR4A1 transcriptional activity is necessary for cell death in this paradigm. Therefore, the cell death pathways activated by both SP/NK_1_R and IGF1R share morphological hallmarks, and the molecular mechanism(s) requires NR4A1. We therefore refer hereafter to cell death induced by either SP/NK_1_R or IGF1R as NR4A1-mediated vesicular cell death.

### NR4A1- mediated vesicular cell death has autophagic features

**Figure 2:**
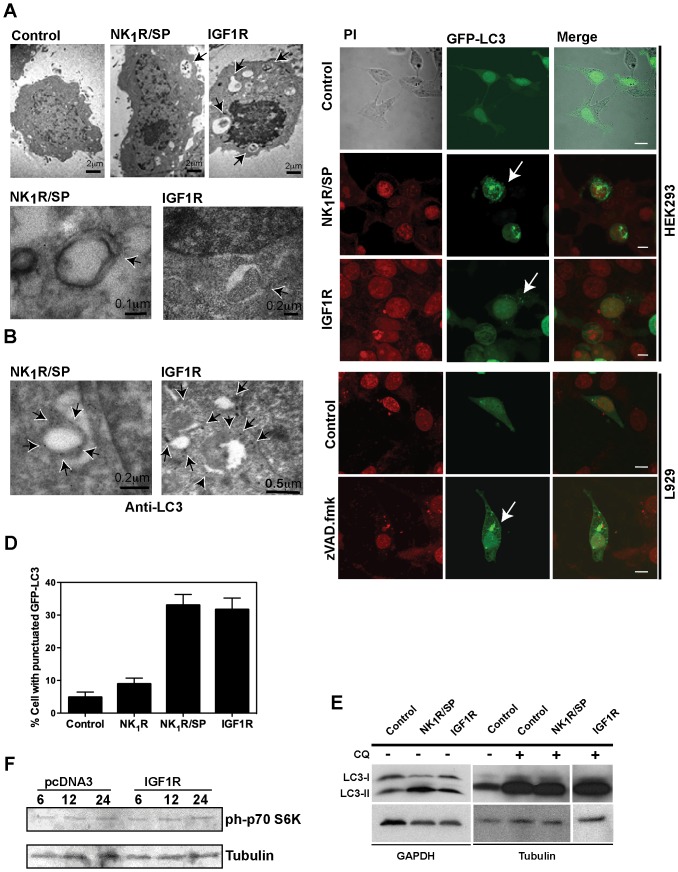
NR4A1-mediated vesicular cell death has autophagic features. **A**, upper panels, presence of vesicles with cytoplasmic content (some marked by arrows) 12 h after cell death induction by either NK_1_R/SP or IGF1R, observed by electron microscopy. Bottom panels, examples of structures resembling autophagosomes about to close (arrows). **B**, Localization of endogenous LC3, examined by immunoelectron microscopy using antibody against LC3, 12 h after cell death induction by either NK_1_R/SP or IGF1R. Arrows indicate examples of LC3 associated with autophagosomes. For **A** and **B**, cells were transfected with empty vector (control), NK_1_R and exposed to SP, or IGF1R, as indicated. **C**, **D**, GFP-LC3 is redistributed during NR4A1-mediated vesicular cell death. HEK293 cells were co-transfected with GFP-LC3 and either the empty vector (control), NK_1_R and exposed to SP, or IGF1R. Examples of cells showing GFP-LC3 punctuated distribution are shown by confocal images in **C**. Percentage of cells with GFP-LC3 re-distribution, among GFP positive cells, are plotted in **D**. 4 fields per treatment were counted, in three independent experiments. Bars represent standard deviation. As a positive reference for autophagy, L929 cells were transfected with GFP-LC3 and exposed or not to caspase inhibitor zVAD.fmk. Nuclei were stained with Propidium Iodide (PI). Scale bar, 5 µm. **E**, LC3-II form accumulates during NR4A1-mediated vesicular cell death. HEK293 cells were transfected with the indicated expression vectors and total protein extracts were collected 24 hr after death induction to detect LC3 by Western blot. GAPDH or Tubulin were detected as loading reference. Autophagic flux did not seem to be impaired, since in the presence of Chloroquine (CQ) LC3-II accumulated furthermore, although there was not a detectable difference between CQ alone and CQ plus cell death inducers (even at lower exposure times). **F**, the autophagy inhibitor kinase mTOR is not activated during IGF1R-induced death. Protein extracts were collected at the indicated times (hr) after transfection with the empty vector pcDNA3 or IGF1R and the phosphorylation of the mTOR target p70 S6K was detected by Western blot. As a loading reference, tubulin was detected in the same blot. As can be observed, the basal level of phosphorylated p70 S6K did not change.

Since both SP/NK_1_R- and IGF1R-induced cell death feature vesicles accumulation, the mechanism to form those vesicles could potentially involve the autophagy core machinery. Although in original studies of these cell death paradigms, double-membrane vesicles (indicative of autophagosomes) were not detected [18], [27], it has now become clear that their occurrence during autophagy is transient. Therefore, their presence may have been missed in those studies, as were performed 24 hr after cell death induction. We repeated the ultrastructural analysis at shorter times, and cells with vesicles containing cytoplasmic componentes 12 h after cell death activation by either SP/NK_1_R or IGF1R were found (Figure 2A upper panel). Also, some structures resembling autophagosomes about to be closed were observed (Figure 2A lower panel).

The basic molecular machinery for the formation of autophagic vesicles involves three functional complexes: 1) the cycling system of Atg9; 2) the PI3K-III complex; and 3) ubiquitin–like systems Atg12-Atg5 and LC3-PE (phosphatidylethanolamine) called LC3-II. This latter conjugation allows a tight association of LC3-II with membranes [34]. In a steady state LC3-I resides in the cytoplasm, but upon autophagy induction it is processed into LC3-II form and translocates to phagophores. In mammals, the processing and re-distribution of LC3-II into the autophagosomes has been used as a marker for autophagosomes and autophagic activity [35].

To further confirm whether the vesicles are derived from autophagosomes, endogenous LC3 was observed by immunoelectron microscopy. Examples of LC3 decorated vesicles are shown in Figure 2B (observe black dots marked by arrows). Also, cells were transfected with a GFP-LC3 fusion protein to follow LC3 localization. As shown in Figure 2C–D, upon cell death induction, approximately 30% of the cells showed GFP-LC3 redistributed into punctuate bodies in both models of cell death. Since punctuated GFP-LC3 was present only in 5% of control cells, the higher incidence of re-distribution observed in NR4A1-mediated vesicular cell death is not an artifact due to GFP-LC3 over-expression. Accordingly, an increase of the processed LC3-II form was detected by Western blot (Figure 2E). Notably, the level of LC3-II increased further in the presence of chloroquine, a lysosomotropic agent that prevents acidification, which leads to inhibition of both fusion of autophagosome with lysosome and lysosomal acidification, with a consequent stop of the autophagic flux. Taken together, these observations indicated that there is an increment in the level of autophagy during NR4A1-mediated vesicular cell death.

One of the main negative regulators of autophagy is the kinase mTOR, an integrator of signals coming from growth factors, nutrients availability, and energetic balance [36]. mTOR is activated by the PI3K-I/Akt pathway, and therefore potentially activated by the expression of IGF1R. Nevertheless, this signaling should not occur to allow the observed increase in autophagy. Indeed, during IGF1R-induced cell death there was no increase of mTOR activity, as indicated by the steady state level of phosphorylation of its substrate, p70 S6K (Figure 2F). Therefore, the signaling pathway activated by IGF1R to induce this form of vesicular cell death prevents the activation of the classical pro-survival pathway.

### NR4A1- mediated vesicular cell death requires the autophagic core machinery

**Figure 3:**
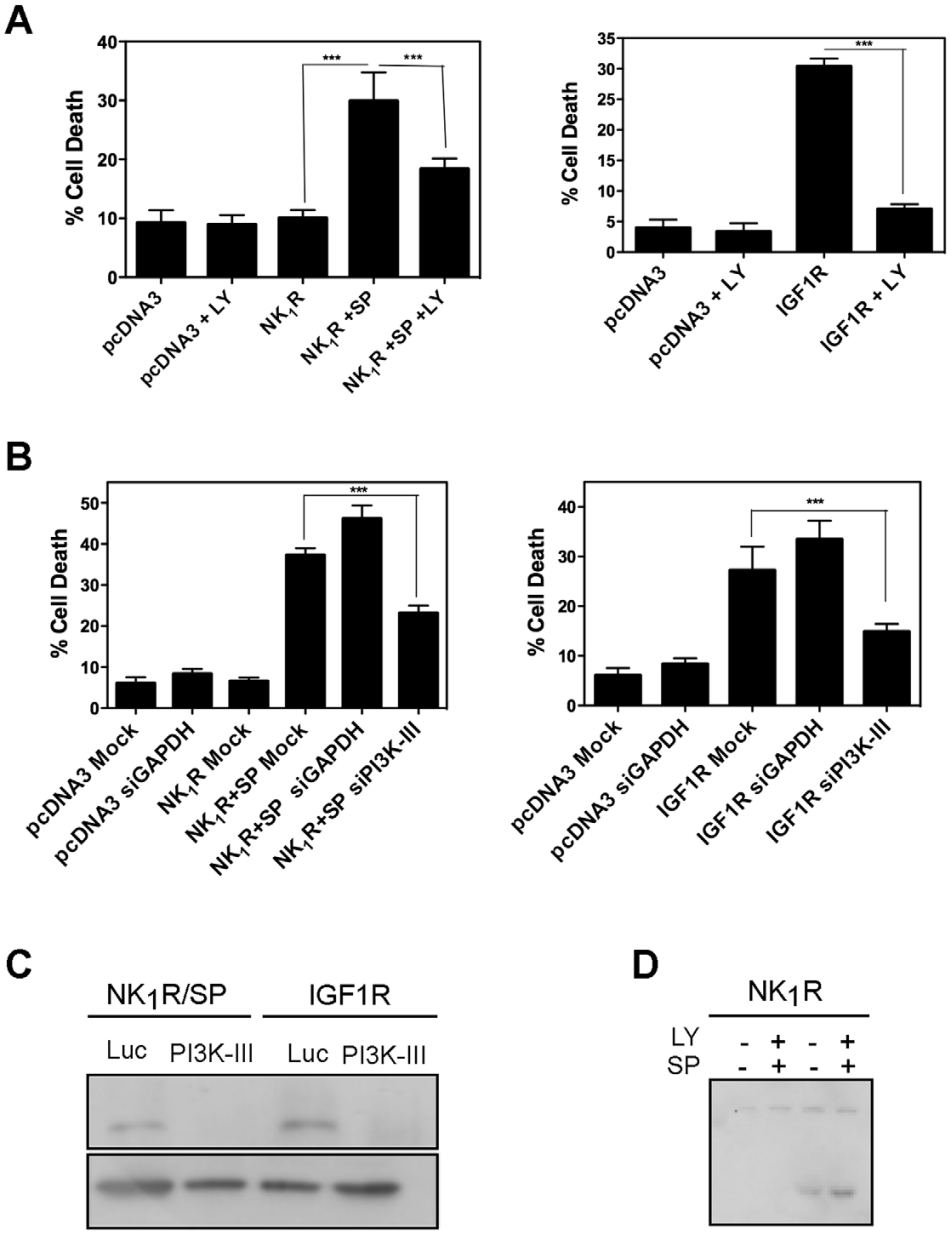
PI3K-III activity is necessary for NR4A1-mediated vesicular cell death. HEK293 cells were transfected with either the empty vector pcDNA3, NK_1_R and exposed or not to SP, or IGF1R as indicated. **A**, Pharmacological inhibition of PI3K with LY294002 reduces cell death. DMSO was added as vehicle control. **B**, Specific down regulation by RNAi of PI3K-III inhibits cell death. HEK293 cells were transfected with the indicated siRNAs. Cells death was determined by trypan blue exclusion 24 hr after induction. Bars represent standard error. ***, p<0.001, n = 4. **C**, Western blot to show the efficiency of RNAi. Luc refer to siRNAs targeting the luciferase gene, as irrelevant siRNA. GAPDH was detected as loading reference. **D**, induction of NR4A1 expression upon cell death activation is upstream of autophagy activation. HEK293 cells were transfected with NK_1_R expression vector and exposed or not SP, and the expression of NR4A1 was monitored by Western blot in the presence or absence of the autophagy inhibitor LY294002, as indicated.

**Figure 4:**
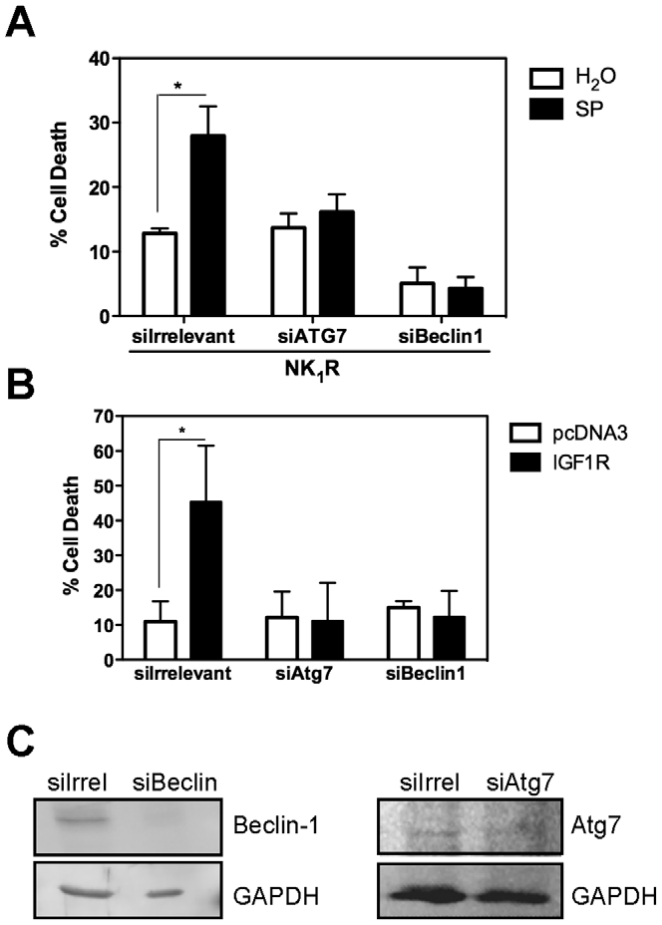
Beclin 1 and Atg7 are necessary for NR4A1-mediated vesicular cell death. **A**, HEK293 cells were transfected with NK_1_R and exposed or not to SP, or **B**, with the empty vector pcDNA3 or IGF1R. Expression of Beclin 1 or Atg7 was blocked by transfecting SMARTpool siRNAs for them in both models. As control, siRNA targeting a sequence not found in the human genome was transfected. Cells death was determined by trypan blue exclusion 24 hr after cell death induction. Bars represent standard error, (n = 4, * p<0.05). **C**, Western blot to show the efficiency of the respective RNAi. GAPDH was detected as loading reference. Atg7 signal was particularly low in these cells, therefore the exposure for this blot was longer and the contrast was increased.

**Figure 5:**
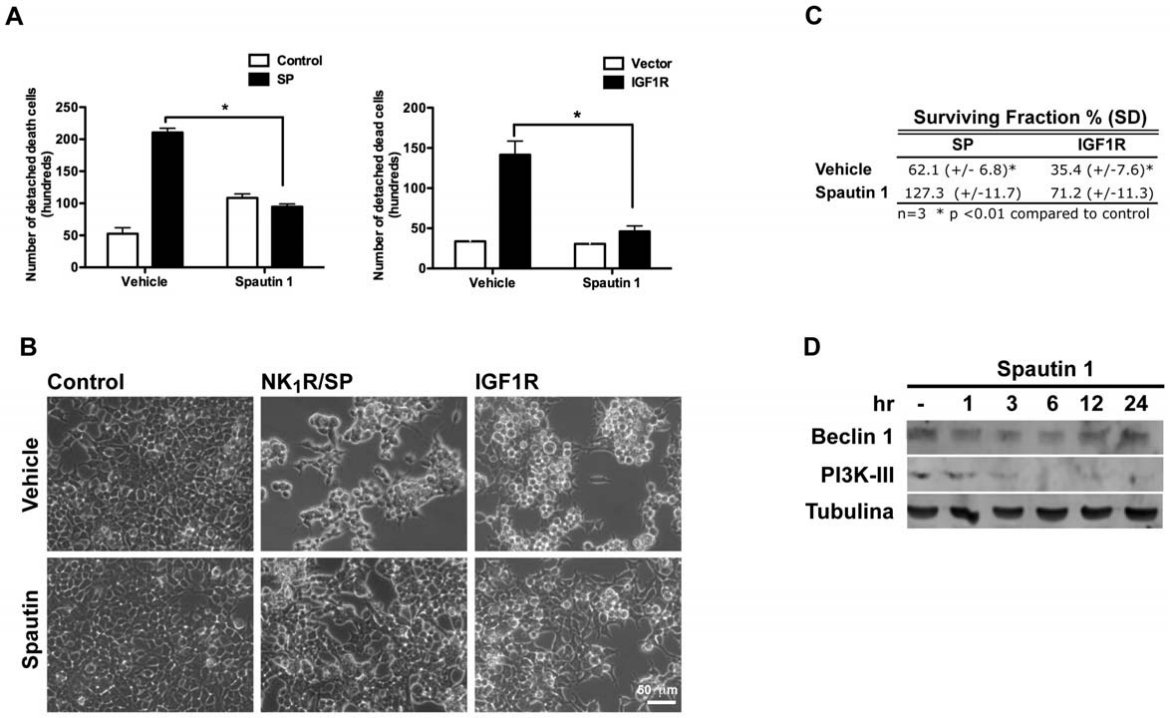
Inhibition of autophagy by Spautin-1 prevents cell death and doubles the clonogenic growth. HEK293 cells were transfected with either the empty vector pcDNA3, NK_1_R and exposed or not to SP, or IGF1R as indicated. Either Spautin-1 or vehicle DMSO were added as indicated. Detached cells were collected from the media and cell death was determined by trypan blue exclusion 24 hr after cell death induction. Bars represent standard error, (n = 3, * p<0.05). **B**, bright field image of the cells that remained attached from the same cultures as in **A**, that were taken for clonogenic growth assays. **C**, Spautin-1 increases the clonogenic growth of cells expressing NK_1_R and exposed to SP, or expressing IGF1R compared with vehicle. Equal number of cells from cultures shown in **B** was seeded, and the number of colonies containing more than 10 cells was scored (details in methods section). The plating efficiency was calculated for control cells and included in the calculation of the survival fraction. **D**, Spautin- 1 promotes the degradation of both Beclin 1 and PI3K-III. Western blot of total protein extracts taken at the indicated times.

In order to determine whether the observed increase in autophagy was necessary to promote cell death, we blocked members of the core complex PI3K-III/Beclin1 either pharmacologically or by RNAi. As shown in Figure 3A, in the presence of the PI3K inhibitor LY294002 there was a reduction of cell death induced by either NK_1_R/SP or IGF1R by 70%. Since LY294002 inhibits several classes of PI3K, specific activity of PI3K-III was downregulated by RNAi. Again, both NK_1_R/SP- and IGF1R- induced cell death was reduced when cells were transfected with siRNA targeting PI3K-III, but not by one targeting an irrelevant gene (GAPDH) (Figure 3B–C). Interestingly, NR4A1 expression occurs upstream of autophagy induction, as NR4A1 expression was not prevented by the autophagy inhibitor LY294002 (Figure 3D). We propose that NR4A1 could modulate autophagy, perhaps converting it into a detrimental process. To further confirm the dependence on autophagy for cell death, we knocked-down by RNAi two other autophagic genes, Beclin 1 and Atg7, and found that it also reduced NK_1_R/SP- or IGF1R-induced cell death (Figure 4). In every case four different targets for siRNA were tested. Spautin-1 is a potent small molecule inhibitor of autophagy, which promotes degradation of PI3K-III and Beclin 1 [37]. To assess whether inhibition of autophagy would only delay cell death, or truly prevent it, we evaluated in both models of NR4A1-mediated vesicular cell death the effect of Spautin-1. We quantified the number of dead cells that detach from the plate and the clonogenic growth potential of the remaining alive cells. As shown in Figure 5A, the presence of Spautin-1 reduced the number of dead cells in both paradigms, measured after 24 hr of cell death induction, while the number of healthy attached cells increased (Figure 5B). To assess the clonogenic growth of the surviving cells, equal number of cells from each condition was seeded and the number of colonies containing more than 20 cells after further 2–5 days was scored. We found that upon Spautin-1 treatment, the number of colonies doubled in both models of cell death; the results are shown in Figure 5C expressed as the surviving fraction with respect to control cells (details in methods section). Spautin-1 promoted the degradation of both Beclin 1 and PI3K-III, accordingly to the reported effect [37]. Interestingly, Beclin 1 expression seemed to be recovered after 24 hr (Figure 5D), and yet cell death was prevented.

Therefore, not only there is an increase in autophagy, but it is also necessary for the progression of cell death, indicating that both NK_1_R/SP and IGF1R activate a signaling pathway that induces NR4A1 expression and leads to cell death by autophagy.

### NR4A1 mediates autophagic cell death induced by caspase inhibition

**Figure 6:**
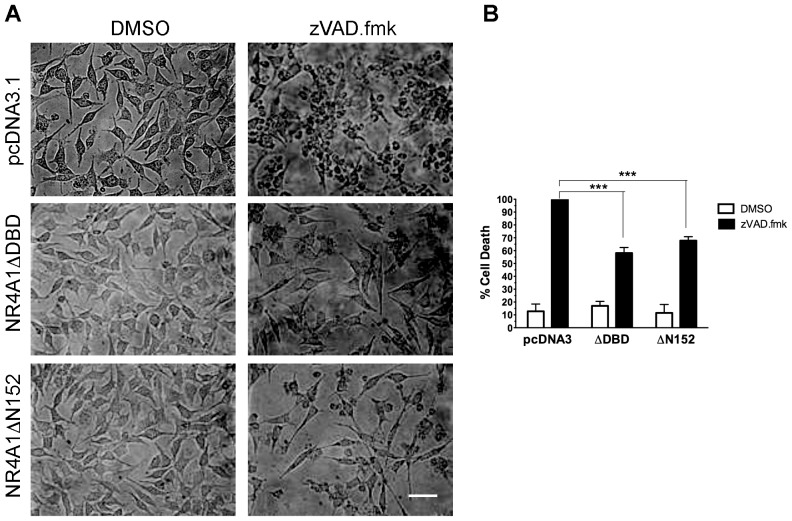
Expression of NR4A1 dominant negative mutants inhibits autophagic cell death induced by caspase inhibition. Mouse fibroblasts L929 were transfected with the indicated expression vectors, and treated or not with the pan-caspase inhibitor zVAD.fmk to induce autophagic cell death. **A**, cells exposed to zVAD.fmk were rounded up unless they expressed the indicated mutants. Scale bar, 50 µm. **B**, cell death determined by trypan blue exclusion 24 hr after treatment. There is a reduction of cell death by the expression of either NR4A1 dominant negative mutant ΔDBD or ΔN152, even though the efficiency of transfection was on average 30%. (n = 3; ***, p<0.001).

Lenardo and collaborators described a form of autophagic cell death in mouse L929 cells activated by inhibition of caspases [17]. We investigated whether NR4A1 activity was necessary for this proved paradigm of cell death by autophagy. As shown in Figure 6, the expression of either of the two NR4A1 dominant negative mutants that inhibited NK_1_R/SP- or IGF1R-induced cell death, also did reduce this model of cell death. Therefore, NR4A1 activity is necessary for cell death dependent on autophagy induced by at least three different stimuli.

### NR4A1 acts as a transcription activator during cell death by autophagy

**Figure 7:**
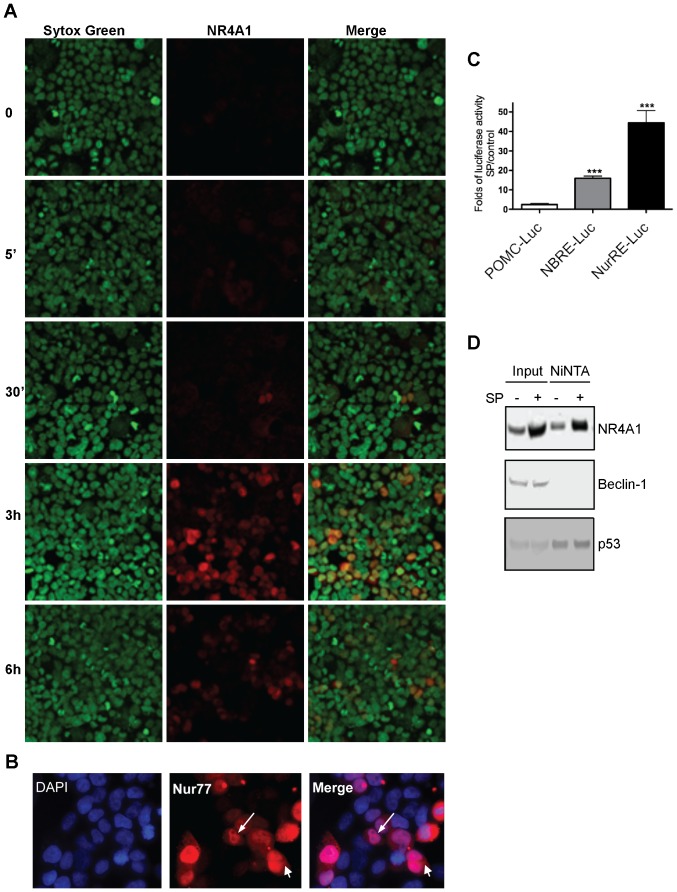
NR4A1 localized mainly in the nucleus during autophagic cell death, and is transcriptionaly competent. HEK293 cells were transfected with NK_1_R and exposed to SP for the indicated time. **A**, Immunofluorescence of NR4A1 (red) showed it mainly in nuclei, which were stained with Sytox Green. 20× magnification. **B**, Examples of cells where NR4A1 (red) was found by immunofluorescence in both the cytoplasm and the nucleus (DAPI) after 3 hr of exposure to SP. 40× magnification. **C**, Luciferase essay to quantify NR4A1 transcriptional activity driven by either NBRE or NuRE containing promotor. Folds of luciferase activity after 6 hr of SP addition with respect to control cells are plotted. Bar represent standard deviation (n = 4, ***, p<0.001). **D**, NR4A1 interacts with p53 but not with Beclin 1. HEK293 cells were transfected with His-tagged NR4A1 and NK_1_R, and exposed or not to SP. NR4A1 was pulled down by NiNTA, and the presence of Beclin 1 or p53 complexed with NR4A1 was analized by Western blot.

NR4A1 can induce apoptotic cell death by at least two different mechanisms: transcriptional and non-transcriptional. Expression of a mutant lacking the DNA binding domain (NR4A1ΔDBD, which is located in the cytoplasm) inhibits the transcriptional mechanism, while it is unable to inhibit cell death in a second, contrasting mechanism, where NR4A1 is transported outside the nucleus and translocated to the mitochondria; in this latter case NR4A1 is transcriptionally inactive, as it does not activate a reporter gene [31]. In contrast, another mutant lacking the trans-activating domain (NR4A1ΔN152, which is located in the nucleus) is able to inhibit mitochondrial targeting of NR4A1 and cell death [31]. Since both mutants inhibited both SP/NK_1_R- and IGF1R-induced death, the mechanism NR4A1 follows to trigger cell death by autophagy could involve either a nuclear or a cytoplasmic function. We investigated the subcellular localization and transcriptional activity of NR4A1 in these cell death paradigms. By immunolocalization, we detected NR4A1 mainly in the nucleus (Figure 7A, B). Although 14% of the cells showed cytoplasmic NR4A1, it did not co-localize with mitochondria or endoplasmic reticulum (data not shown), unlike what has been observed during apoptosis [31], [32]. NR4A1 can bind to two different response elements: NuRE, where it binds as a homodimer or heterodimer; and NBRE, where it binds as a monomer. Using a luciferase reporter containing either NuRE or NBRE, we observed NR4A1 transcription activity 6 hr after cell death activation, with a stronger response of NuRE (Figure 7C). This finding suggests the possibility that NR4A1 mediates cell death by autophagy at least in part by acting as a dimeric transcription factor. On the other hand, it has been shown that NR4A1 has the ability to interact with the anti-apoptotic protein Bcl2, which interacts with Beclin-1 and prevents autophagy [38]. We tested, then, whether NR4A1 could interact with Beclin-1 complex to promote autophagy. As shown in Figure 7D, it was not the case. Interestingly, NR4A1 interacts instead with the tumor suppressor p53, a protein able to induce autophagy by several mechanisms, including the transcription of target genes like AMPK [39] or DRAM [40], among other mechanisms. We found that neither AMPK nor DRAM increased during NK_1_R/SP induced death (data not shown).

## Discussion

In this report we show that two evolutionarily different receptors, NK_1_R—belonging to the G-protein coupled receptor family—and IGF1R—a tyrosine kinase receptor—both induce cell death by autophagy, as proteins essential for the autophagic core machinery were required. Inhibition of the NR4A1 activity by the expression of dominant negative mutants also prevented another form of cell death by autophagy, induced by caspases inhibition in L929 cells. Since NR4A1 can heterodimerize with the other members of the family Nor1 and Nurr1, the dominant negative mutants used in this study can potentially inactivate them as well. Nevertheless, we found no expression of Nor1 (data not shown), and although Nurr1 mRNA was detected in a microarray cDNA hybridization analysis, its specific knockdown is necessary to determine whether Nurr1 also has an active role in promoting this type of cell death. We focus on NR4A1 in particular because: 1) the known functions of Nurr1 does not support a pro-autophagic cell death activity; 2) when NR4A1 activity was specifically inhibited by RNAi, cell death induced by NK_1_R/SP was impaired; 3) NR4A1 is activated during LPS-activated macrophage death, which is a caspase-independent [4], but autophagy-dependent death program [41].

On the other hand, we observed NR4A1 activity is not necessary for necroptosis (data not shown), a different form of non-apoptotic cell death that displays autophagic features, but which is independent of the autophagic core machinery [42]. Therefore, NR4A1 can modulate either apoptosis or cell death by autophagy, but not other forms of cell death independent of caspases and autophagy. Considering our data and literature all together, we propose that NR4A1 is a common mediator of cell death by autophagy.

Autophagy is a catabolic process that helps to maintain the cells' health. But when autophagy is sustained or inappropriately induced it may kill the cell by an unknown mechanism. Specific proteins may be preferentially targeted for autophagic degradation [43] or mitochondrial fission could be increased to stimulate excessive mitophagy [44]. This suggests the possibility of a selective degradation of regulatory molecules or organelles that are essential for survival. Alternatively, a loss of lysosomal integrity associated with an impairment of the autophagic flux could switch autophagy into a detrimental process [45]. Therefore, molecules that modulate either mitochondrial dynamics, the specificity of the cargo, or lysosomal permeability, could potentially transform the pro-survival effect of autophagy into a pro-death effect. It will be interesting to study whether NR4A1 is one of such molecules, and whether either of these mechanisms occurs. The increment in LC3-II we observed in this study (Figure 2E) might reflect a combination of an induction of autophagy with an inhibition of the autophagic flux, since there was not a substantial difference in the amount of LC3-II with chloroquine alone than with chloroquine plus cell death inducers. Nevertheless, we do not rule out that autophagy can still be induced, because there was a huge increase in the level of LC3-II with chloroquine, compared with cell death inducers. This latter observation suggests that a significant amount of LC3-II is being degraded during NR4A1-mediated cell death.

Unlike in some other paradigms of autophagic cell death, the pathway of cell death that we describe here occurs in cells competent to undergo apoptosis, as shown in previous studies [18], [27]. This is not unprecedented, however, since cell death by autophagy in apoptosis competent cells has also been observed elsewhere, like in astrocytes under hypoxic conditions and in MEFs, mediated by apoL1, a BH3-only pro-death protein [46]. Interestingly, it has recently been demonstrated that the high mortality associated to influenza infection by H5N5 is due to its ability to induced autophagic cell death in lung cells *in vivo*, in wild type and therefore apoptotic competent mice [47].

NR4A1 is a multifunctional protein with the ability to modulate proliferation, differentiation and apoptosis. Here we show that NR4A1 can, in addition, promote cell death by autophagy. How NR4A1 can mediate alternative forms of cell death under different conditions deserves further investigation. NR4A1 seems to be a mediator of programmed cell death, bridging apoptotic and non-apoptotic pathways. The previously demonstrated interplay between apoptosis and autophagy [15] is compatible with the notion that NR4A1 may be pivotal in these two pathways. Bcl-2 interacts with Beclin 1 through their BH3 domains, preventing autophagy [38]. Since NR4A1 is able to interact with Bcl-2, perhaps NR4A1 may displace Bcl-2, releasing Beclin 1 to promote autophagy. We determined here that NR4A1 did not interact with Beclin 1, although Beclin 1 was necessary for cell death.

NR4A1 activity sometimes depends on its nuclear-to-cytoplasmic translocation. It can function as a transcriptional regulator or as a protein interactor in cytoplasmic compartments [31], [32] where it modulates the function of Bcl2 family members [33]. In colon cancer cells in response to the short chain fatty acid anion butyrate, the nonsteroidal anti-inflammatory drug sulindac, or the chemotherapeutic drug 5-fluorouracil, NR4A1 induces apoptosis by nuclear-cytoplasmic translocation but without direct mitochondrial targeting, although Bax is redistributed to mitochondria and induces cytochrome c release [48]. In the case of SP/NK_1_R-induced death, we found that NR4A1 is translocated to the cytoplasm, although it is also retained in the nucleus and remains transcriptionally competent. This finding, together with the ability of the dominant negative mutant NR4A1ΔDBD to prevent the three paradigms of cell death by autophagy, suggests that NR4A1 may effects the switch from protective to destructive autophagy by transcribing pro-autophagic genes, and may also interact with some of the resultant proteins to mediate its non-transcriptional effects. Specific target genes are currently under investigation and further experiments are needed to test this hypothesis. Autophagy has a contrasting role in carcinogenesis. On one hand positive regulators of autophagy are tumor suppressors. 45–75% of cases of human prostate, breast and ovarian cancers present monoallelic mutations of the *Beclin-1* gene. On the other hand, certain autophagy inhibitors also confer tumor suppression, perhaps because autophagy removes damaged organelles, growth factors or drugs, thus functioning as a cytoprotective mechanism for cancer cells. Interestingly, NR4A1 has contrasting roles in cancer as well. On one hand, NR4A1 induces cell death; on the other hand, NR4A1 silencing induces apoptosis in several cancer lines [49]. Therefore, understanding the molecular basis of cell death mediation by NR4A1, and its ability to modulate both apoptotic and autophagy-dependent cell death, should provide novel insights and targets for therapeutic intervention.

Dysfunctions of autophagy are involved in several pathologies besides neurodegeneration and cancer, like liver and muscle disorders, or pathogen invasion. Therefore, understanding its molecular regulation has a wider potential impact on medicine.

## Materials and Methods

### Cell culture, plasmids transfection and cell death evaluation

Human embryonic kidney 293 cells were grown in high glucose DMEM (Invitrogen, Carlsbad, CA) supplemented with 10% fetal bovine serum (Sigma, St. Louis, MO) and penicillin/streptomycin 100 U/ml (Invitrogen, Carlsbad, CA). The cultures were incubated at 37°C in 95% air and 5% carbon dioxide with 95% humidity. Transient transfection was performed by calcium phosphate/DNA co-precipitation. Briefly, 2×10^5^ cells/well were seeded into 35 mm wells 16 hr prior to transfection. Transfection solution: 5 µg of DNA with 250 mM CaCl_2_ in 50 µl were incubated 10 minutes at room temperature. Then 50 µl of 2X HEPES Buffer Saline (HBS: 230 mM NaCl, 10 mM KCl, 1.5 mM Na_2_HPO_4_, 12 mM Dextrose, 50 mM HEPES, pH 6.95) were added and incubated 2 min. at 37°C. Transfection solution was left during 12–15 hours. After 24 hr, 100 nM SP (SIGMA) was added when necessary. Expression of each construct in the transient transfections was determined by Western blot or immunofluorescence. The plasmids pcDNA3.1-NK_1_R and pcDNA3.1-IGF1R-IC have already been described [18], [27]. Dr. Noboru Mizushima (Tokyo Medical and Dental University, Japan) kindly shared the pCAG-GFP-LC3 construct and Dr. Jacques Drouin (Laboratoire de Génétique Moléculaire, Institut de Recherches Cliniques de Montréal, Canada) kindly provided POMC-Luc; NBRE-POMC-Luc and NuRE-POMC-Luc reporter plasmids. Transient transfection efficiencies were in all cases >80%. Cell death was determined by trypan blue exclusion. PI3K inhibitor LY294002 (Calbiochem #440202, Los Angeles, CA, USA) was used at 5 µM. Autophagy inhibtor Spautin-1 (Cellagen Technology, San Diego, CA, USA) was used at 10 µM. The software PRISM 5.0 (GraphPad Software, La Jolla, CA, USA) was used for the one way ANOVA statistical analysis, and the p values between indicated treatments in each figure were calculated by Bonferroni's Multiple Comparison Test.

To assess clonogenic ability, cells were treated as described above for cell death evaluation and re-plated after 24 hr. Equal number of cells was taken from each treatment and seeded 100 up to 10^4^ cells per well (6 well plates) for 2–5 days to allow colonies to form; cells were washed with PBS, fixed and stained with a mixture of 6% glutaraldehyde and 0.5% crystal violet over night, rinsed with tap water and dried at room temperature. Colonies with more than 20 cells were counted. The plating efficiency (PE) was calculated for control cells as the ratio of the number of colonies to the number of cells seeded. The surviving fraction (SF) is the number of colonies that arise after treatment of cells, expressed in terms of PE: SF = no. of colonies formed after treatment/(no. of cells seeded X PE) [50].

Murine L929 cells (kindly provided by Dr. Alejandro Zentella at Departamento de Medicina Genómica y Toxicología ambiental, Instituto de Investigaciones Biomédicas, UNAM, Mexico) [51] were transfected using Lipofectamine 2000 (Invitrogene, Carlsbad, CA, USA) according to manufacturer instructions. Briefly, 3.5×10^5^ cells/well were seeded into 35 mm wells 24 hr before transfection. For each well, 4 µg DNA: 12 µl Lipofectamine ratio was used. The percentage of cells with punctuated LC3-GFP out of 100 GFP expressing cells from each condition in each experiment were calculated. Cells were not fixed. Fluorescent cells were detected using either a Nikon Eclipse TE300 fluorescence microscope, or an Axiovert 200M (Carl Zeiss) confocal microscope.

### Electron Microscopy and immunoelectron microscopy

Cell pellets were fixed in 4% paraformaldehyde/2.5% glutaraldehyde for 1 hr at room temperature; then it was washed two times (10 min each) in PBS and stained with 1% osmium tetraoxide for 1 hr at 4°C, followed by two washes with PBS and water. Total dehydration was made in ethanol (graded 50–100%) and propylene oxide. Pellets were embedded in epoxy resin and cut into 70 nm sections. For immunoelectron microscopy, cell pellets were fixed with 4% paraformaldehyde/0.1% glutaraldehyde for 30 min at 4°C. After ethanol dehydration (graded 70–100%) pellets without osmication were embedded in LR White. Ultra-thin sections were mounted on nickel grids, blocked with 5% fat free Milk in TBST for 15 min and probed with a 1∶10 dilution of anti LC3 (Cell Signaling Technology Inc., Danvers, MA, USA) at 4°C over night. A 1∶10 dilution of colloidal gold-conjugated secondary antibody (GAR Auroprobe, Amersham) was incubated for 2 hr at room temperature. Then sections were washed with TBST and PBS, post-fixed with 1% glutaraldehyde in PBS, thoroughly washed and stained with 2% aqueous uranyl acetate. A control without first antibody was included. Electron microscopy was performed at 80 kV on a Zeiss EM900 Transmission Electron Microscope. Images were recorded with a Gatan Dual Vision CCD 300W camera (Gatan, Pleasanton, CA).

### Western blot and immunofluorescence analysis

For Western, the transfected human embryonic kidney 293 cells were washed with cold PBS and homogenized in lysis buffer (150 µM NaCl, 1% Triton X-100, 50 µM Tris HCl pH 8.0, proteinase inhibitors cocktail (Roche Diagnostic Corporation, Indianapolis, IN, USA)). Cytoplasmic extracts were collected after a 10 min. centrifugation at 14,000 rcf. Protein was quantified by Bradford assay and electrophoresis of equal amounts of total protein was performed on SDS-polyacrylamide gels. Separated proteins were transferred to polyvinylidene fluoride membranes at 4°C. Membranes were probed with a 1∶1000 dilution of anti-LC3 (Cell Signaling Technology Inc., Danvers, MA, USA); 1∶8000 dilution of anti-GAPDH (Research Diagnostics, Flanders, NJ, USA); 1∶500 dilution of anti-PI3K-III (Abcam, Cambridge, UK); 1∶1000 dilution of anti-Beclin 1 (Santa Cruz Biotechnology, Santa Cruz, CA, USA); 1∶1000 dilution of anti-Atg7, anti phospho-p70 S6K (Thr389) and anti p53 (Cell Signaling Technology Inc., Danvers, MA, USA); 1∶7000 dilution of anti-tubulin (Abcam, Cambridge, MA, USA). The membranes were incubated in the appropriate horseradish peroxidase-coupled secondary antibody for 1 hr followed by enhanced chemiluminescence detection (Amersham, Arlington Heights, IL). Alternatively, appropriate infrared dye-coupled secondary antibodies (anti-rabbit IRDye800 and anti-mouse IRDye700, Rockland, Gilbertsville, PA, USA) were used and the blots were scanned in an Odyssey Imager (LI-CORE Biosciences, Lincoln, Nebraska, USA). A 6xHis tagged NR4A1 protein was purified by NiNTA affinity column following provider instructions (Qiagen, Hilden, Germany), separated by SDS-PAGE and blotted to detect Beclin-1 or p53 co-purification. For immunofluorescence, cells were seeded into Lab-Tek CC2 treated slide chambers (Nalgene Nunc International, Napeville, IL, USA); after washing with PBS cells were fixed with 4% paraformaldehyde/PBS for 10 min; then washed with PBS and permeabilized with 0.2% Triton X-100/PBS for 10 min. Afterwards cells were washed with PBS, pre-incubated 30 min. with 4% BSA/PBS and 4% serum goat and washed again with PBS. Anti-NR4A1 (M-210 Santa Cruz Biotechnology, Santa Cruz, CA, USA) was diluted 1∶200 in 2%BSA/PBS and incubated over night at 4°C. After washing with PBS cells were incubated with anti-rabbit coupled to Alexafluor 594 (Invitrogene, Carlsbad, CA) diluted 1∶1000 in 2%BSA/PBS for 30 min at room temperature. Then, cells were treated with 10 µg/ml RNAse for 30 min at 37°C and washed again with PBS. Cells were counterstained with 10 ng/ml Sytox Green (Invitrogene, Carlsbad, CA) and mounted for microscope observation on an Axiovert 200M (Carl Zeiss) confocal microscope.

### RNAi

Two non-overlapping regions (776–794 and 1150–1168 positions in GenBank NM_002647) were targeted for PI3K-III down regulation, and the corresponding siRNAs were simultaneously transfected. These siRNAs were generated by *in vitro* transcription using the Silencer siRNA Construction Kit (Ambion, Austin, TX, USA), following the manufacturer's instructions. siRNA targeting GAPDH was synthesized using the oligonucleotides provided by the kit. In addition, a siRNA control sequence was synthesized targeting the region 153–173 of firefly luciferase gene from the plasmid pGL2-control (GenBank X65324). siRNAs for down regulation of Beclin-1 and Atg7 were purchased as SMARTpool from Dharmacon (Lafayette, CO, USA).

siRNA transfection: Human embryonic kidney 293 cells (10^5^ cells per well in 12-well plates) were grown in high glucose DMEM supplemented with 10% fetal bovine serum (Sigma, St. Louis, MO), with no antibiotics for 16 hr. The siRNA specific for each target gene was transfected with Lipofectamine 2000 reagent (Invitrogene, Carsband, CA, USA) according to the manufacturer's instructions, using 3 µg siRNA: 6 µl Lipofectamine 2000 ratio. After 4 hr of incubation, plasmids were transfected. To estimate the efficiency of the transfection the siRNAs for luciferase (as a sequence irrelevant for the mammalian genome) was chemically synthesized and fluorescently labeled with FITC (Xeragon-Qiagen, Valencia, CA, USA).

### Transcription Assays

Human embryonic kidney 293 cells were seeded in 12-well plates (1.5×10^5^ cells/well). Transient transfections were performed by calcium phosphate/DNA co-precipitation of 1 µg of NK_1_R; 1 µg of reporter plasmid (either POMC minimal promoter-Luciferase (lacks responsive elements); NBRE-Luciferase or NurRe-Luciferase); and 200 ng of pCH110 (encoding *lac Z*) to normalize transfections. Twenty four hours after transfection the cells were incubated or not with 100 nM SP for 5 h. Luciferase and ß-galactosidase activities were determined using the Dual Ligth System (Applied Biosystems), according to manufacturer's instructions, and a Monoligth 2010 (Analytical Luminescence Laboratory) luminometer.
