# Correction: The Nuclear Receptor NR4A1 Induces a Form of Cell Death Dependent on Autophagy in Mammalian Cells

**DOI:** 10.1371/journal.pone.0118718

**Published:** 2015-03-13

**Authors:** Jimena Bouzas-Rodríguez, Gabriela Zárraga-Granados, María del Rayo Sánchez-Carbente, Rocío Rodríguez-Valentín, Xicotencatl Gracida, Dámaris Anell-Rendón, Karen S. Poksay, David T. Madden, Luis Covarrubias, Susana Castro-Obregón

Dr. Karen S. Poksay is not included in the author byline. She should be listed as the seventh author and affiliated with Buck Institute for Research on Aging, Novato, California, United States of America. The contributions of this author are as follows: Performed the experiments.

Dr. David T. Madden is not included in the author byline. He should be listed as the eighth author and affiliated with College of Pharmacy, Touro University California, Vallejo, California, United States of America. The contributions of this author are as follows: Analyzed the data and contributed reagents/materials/analysis tools.
